# Surveillance of cirrhosis for hepatocellular carcinoma: a cost–utility analysis

**DOI:** 10.1038/sj.bjc.6604301

**Published:** 2008-04-01

**Authors:** J Thompson Coon, G Rogers, P Hewson, D Wright, R Anderson, S Jackson, S Ryder, M Cramp, K Stein

**Affiliations:** 1 Peninsula Technology Assessment Group (PenTAG), Peninsula College of Medicine and Denistry, (Universities of Plymouth and Exeter), Noy Scott House, Barrack Road, Exeter EX2 5DW, UK; 2 School of Mathematics and Statistics, University of Plymouth, Drake Circus, Plymouth PL4 8AA, UK; 3 Plymouth Hospitals NHS Trust, Derriford Hospital, Derriford Road, Plymouth PL6 8DH, UK; 4 Wolfson Digestive Diseases Centre, Queen's Medical Centre, Nottingham University Hospital NHS Trust, Derby Road, Nottingham NG7 2UH, UK

**Keywords:** cost-effectiveness, cost–utility, modelling studies, hepatocellular carcinoma, cirrhosis, surveillance

## Abstract

Using a decision-analytic model, we evaluated the effectiveness and cost-effectiveness of surveillance for hepatocellular carcinoma (HCC) in individuals with cirrhosis. Separate cohorts with cirrhosis due to alcoholic liver disease, hepatitis B and hepatitis C were simulated. Results were also combined to approximate a mixed aetiology population. Comparisons were made between a variety of surveillance algorithms using *α*-foetoprotein (AFP) assay and/or ultrasound at 6- and 12-monthly intervals. Parameter estimates were obtained from comprehensive literature reviews. Uncertainty was explored using one-way and probabilistic sensitivity analyses. In the mixed aetiology cohort, 6-monthly AFP+ultrasound was predicted to be the most effective strategy. The model estimates that, compared with no surveillance, this strategy may triple the number of people with operable tumours at diagnosis and almost halve the number of people who die from HCC. The cheapest strategy employed triage with annual AFP (incremental cost-effectiveness ratio (ICER): £20 700 per quality-adjusted life-year (QALY) gained). At a willingness-to-pay threshold of £30 000 per QALY the most cost-effective strategy used triage with 6-monthly AFP (ICER: £27 600 per QALY gained). The addition of ultrasound to this strategy increased the ICER to £60 100 per QALY gained. Surveillance appears most cost-effective in individuals with hepatitis B-related cirrhosis, potentially due to younger age at diagnosis of cirrhosis. Our results suggest that, in a UK NHS context, surveillance of individuals with cirrhosis for HCC should be considered effective and cost-effective. The economic efficiency of different surveillance strategies is predicted to vary markedly according to cirrhosis aetiology.

Hepatocellular carcinoma (HCC) occurs mainly in cases of cirrhosis, which, in turn, tend to be secondary to either alcoholic liver disease (ALD) or infection with hepatitis B or C viruses (HBVs/HCVs). Symptomatic HCC typically presents late with a bleak prognosis, whereas HCCs detected during formal surveillance are smaller, more likely to be uninodular and more commonly amenable to curative treatment (De Masi *et al*, 2005). For this reason, UK (Ryder, 2003), European (Bruix *et al*, 2001) and American (Bruix and Sherman, 2005) clinical guidelines recommend routine surveillance for HCC among individuals with cirrhosis, and approximately three-quarters of UK gastroenterologists undertake such a programme, mostly using a combination of periodic serum *α*-foetoprotein (AFP) testing and ultrasound (US) (Lai *et al*, 2002).

However, consensus has not been reached as to the optimal surveillance strategy. We therefore developed a decision-analytic model to assess the effectiveness and cost-effectiveness of a range of different surveillance strategies in the United Kingdom. We considered populations with cirrhosis secondary to HBV or HCV infection, and as a result of ALD.

## MATERIALS AND METHODS

### Overview of model

We developed a state-transition (Markov) model using TreeAge Pro™ 2005 (TreeAge Software, Williamstown, MA, USA) to compare alternative surveillance strategies. In this approach, disease progression is modelled as movement between different health states over time. Time is modelled as a series of fixed cycles, in this case 1 month, with probabilities of movement between states calculated per cycle. Costs and utility values are attached to each state, and the differences between the aggregated costs and health outcomes in each simulation are used to estimate the cost-effectiveness of surveillance, expressed as incremental cost per quality-adjusted life-year (QALY) (Sonnenberg and Beck, 1993). Costs and QALYs were discounted at 3.5% per year (HM Treasury, 2003). The perspective of the analysis is that of the UK NHS.

### Simulated populations

The population of interest is people with compensated cirrhosis aged 70 years or less with no pre-existing medical conditions that might preclude treatment with liver transplantation (OLT) or hepatic resection (including current alcohol or intravenous drug use). The model considers three cirrhosis aetiologies (ALD, HBV and HCV). Results were also combined to simulate a mixed aetiology cohort, consisting of the following proportions (based on an average of estimates provided by gastroenterologists in several centres around the United Kingdom): 57.6% ALD, 7.3% HBV and 35.1% HCV.

### Model structure

**Figure 1:**
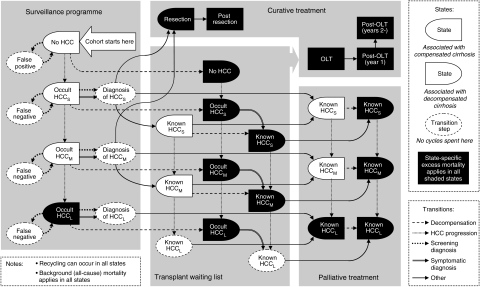
Influence diagram illustrating the natural history and treatment pathways simulated in the model.

The structure of the model is shown in Figure 1. For the natural history component of the model, we defined three classes of HCC: small (<2 cm in diameter), medium (2–5 cm) and large (>5 cm). Tumour characteristics in terms of detectability and treatability are reflected in the transition probabilities. The following simplifying assumptions were made: (i) progression from cirrhosis to decompensated cirrhosis is irreversible; (ii) the rate of incidence of HCC is the same in compensated and decompensated livers; (iii) tumour diameter is used as a surrogate index of all characteristics of tumour progression (multifocal tumours are not modelled separately, since tumour nodularity is heterogeneous in the populations from which parameters have been drawn; therefore, what our model defines as ‘medium’ tumours would, in real-world practice, include those with multiple, small nodules that would not preclude transplantation, and a ‘large’ tumour may be thought to include those which are diffuse in nature); (iv) the presence of an HCC is only associated with additional mortality risk when it becomes ‘large’ (at which point it is also very likely to become symptomatic) and (v) compensated cirrhosis is not subject to an excess mortality rate, as the primary causes of death in these individuals are those already accounted for in the model (i.e., progression to decompensation and/or HCC).

**Figure 2:**
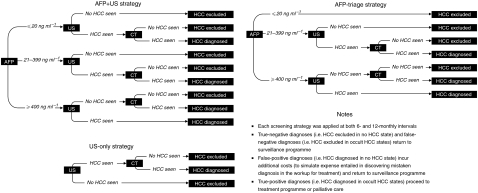
Decision trees illustrating the three screening algorithms investigated in the model.

The surveillance programme is superimposed onto the disease process. We simulated six different surveillance regimes, consisting of AFP-led, US-led and combined screening at 6- or 12-monthly intervals (Figure 2). These were based on European guidelines, which recommend that the diagnosis of HCC be based on two coincident imaging techniques (Bruix *et al*, 2001). For comparison, we also modelled an arm simulating no surveillance. The possibility of incidental/symptomatic presentation of HCC is modelled at all stages of disease. All confirmatory imaging is by CT scan. We assumed 100% compliance with the surveillance programme in the base-case analysis.

We modelled a mixed treatment approach using OLT and resection. People can enter the OLT waiting list following diagnosis of either a surgically treatable HCC or decompensated cirrhosis. Each person is as likely to receive a liver as any other, regardless of the reason for listing. While on the waiting list, people are subject to the same natural history process as those prelisting, and no ‘bridging’ therapies are simulated. People who undergo successful surgical treatment enter a simplified disease process in which excess mortality rates and associated costs and utilities encompass the spectrum of possible post-treatment experiences. There is no waiting list for liver resection. Some people are deemed unsuitable for either surgical treatment. A proportion of people with surgically untreatable small and medium-sized tumours receive palliative treatment with radiofrequency ablation and percutaneous ethanol injection. On progression to ‘terminal HCC large’, an excess mortality with associated costs and utilities is applied that reflects the palliation provided by transarterial chemoembolisation for a proportion of people.

### Parameters

Model parameters are listed in Tables 1, 2 and 3. Estimates were obtained from literature searches in a range of electronic databases (full details and strategies available from the authors). We sought data that fulfilled the following criteria: large, recent studies of UK patients with a diagnosis of cirrhosis (with details of aetiology). For parameters in which there were no UK-based studies available, we sought data from countries with a similar disease profile. Cost data were obtained from national (UK NHS; Department of Health (DoH), 2005) sources where available, supplemented by data from a recent UK-based observational study of patients with HCV (Wright *et al*, 2006). Historical values were inflated to 2004 prices.

### Analysis of uncertainty

Extensive one-way sensitivity analyses were undertaken to explore, which of the input parameters had the greatest impact on results. For simplicity, these were performed using a single-core comparison: 6-monthly AFP+US *vs* no surveillance. Owing to the paucity of reliable estimates for US sensitivity and the possibility that these estimates do not accurately reflect current practice, we examined the impact of simultaneously varying the sensitivity of US for detecting tumours over a range of correlated values from 5 to 50, 10 to 75 and 50 to 100% for small, medium and large tumours, respectively. We also performed scenario analyses testing less optimistic assumptions about patient compliance.

Probabilistic sensitivity analysis was also undertaken. Ten thousand Monte Carlo simulations per aetiology were run, with key input values randomly drawn from probabilistic density functions in each iteration. Distributions can be obtained from the authors.

## RESULTS

### Effectiveness of surveillance

Table 4 summarises the effectiveness of each surveillance strategy in the mixed aetiology cohort. The 6-monthly AFP+US was most effective across all outcomes, more than tripling the number of HCCs diagnosed while operable, and almost halving the number dying from HCC, when compared with no surveillance. However, the cheapest strategy, annual AFP-triage, still achieved substantial gains: for example, more than doubling the number of operable HCC found and increasing the number of small tumours found more than six-fold.

### Cost–utility of surveillance

Cost–utility results are shown in Table 5. In an incremental analysis, neither of the US-only strategies would be considered (since they are both slightly less effective and more costly than surveillance at the same frequency with AFP-triage). Therefore, in the mixed aetiology cohort the cheapest surveillance strategy is annual AFP-triage, with incremental cost-utility of £20 700 per QALY. Doubling the frequency of surveillance would increase the mean number of QALYs by 0.035 at a cost of £1000 each, giving an incremental cost-effectiveness ratio (ICER) of £27 000 per QALY gained.

### Deterministic sensitivity analyses

The cost–utility estimates appear to be most sensitive to changes in tumour growth rate, mortality following OLT and excess mortality associated with undiagnosed large tumours. Quality of life in compensated cirrhosis and following OLT are also important, as are costs associated with US and OLT. When the cirrhosis aetiologies are considered separately, the mean age at diagnosis in individuals with HBV-related cirrhosis becomes an important variable.

Increases in US sensitivity lead to improved effectiveness in all surveillance strategies. At both annual and 6-monthly frequencies, US-led surveillance becomes more effective than AFP-triage surveillance when it can be assumed that US is at least sensitive enough to detect one in five small tumours, one in three medium tumours and two in three large tumours. However, when costs are also considered, it is only in the HBV cohort that US becomes more cost-effective than AFP-triage surveillance at the same frequencies.

When we examined imperfect patient compliance with surveillance, we found a noticeable reduction in effectiveness. However, there was a commensurate reduction in costs, so incremental cost-effectiveness results were not greatly altered.

The model is extremely sensitive to the discount rate applied (3.5% per year for both costs and utilities in the base case). The ICER for 6-monthly AFP+US compared with no surveillance ranges from £19 400 per QALY if no discounting is applied to £35 800 per QALY if rates of 6% are used for both costs and utilities.

### Probabilistic sensitivity analysis

**Figure 3:**
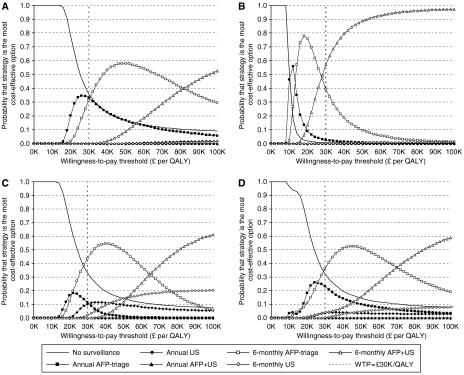
Cost-effectiveness acceptability curves, showing relative probability of maximal cost-effectiveness among surveillance strategies. (**A**) ALD-related cirrhosis; (**B**) HBV-related cirrhosis; (**C**) HCV-related cirrhosis; (**D**) mixed aetiology cohort (weighting: 57.6% ALD; 7.3% HBV; 35.1% HCV). Maximal cost-effectiveness reflects the proportion of Monte Carlo simulations (10 000 per aetiology) in which each strategy generated the highest net monetary benefit.

Figure 3 shows cost-effectiveness acceptability curves (Fenwick *et al*, 2001; Fenwick *et al*, 2004) for ALD, HBV, HCV and the mixed cohort. These graphs show the probability that each strategy would be considered the most cost-effective (in terms of highest net monetary benefit) at different levels of willingness to pay for a QALY.

At a willingness-to-pay threshold of £30 000 per QALY, the most intensive surveillance protocol simulated (6-monthly AFP+US) is only likely to be considered cost-effective in individuals with HBV-related cirrhosis. In those with HCV-related cirrhosis, 6-monthly AFP-triage is more likely to be considered cost-effective; indeed, willingness to pay would have to rise to around £65 000 per QALY before 6-monthly AFP+US becomes most likely to be considered cost-effective in this population. In individuals with ALD-related cirrhosis, there is uncertainty about which strategy would be most cost-effective at a willingness-to-pay threshold of £30 000 per QALY, with no surveillance, annual AFP-triage and 6-monthly AFP-triage having approximately equal likelihood of maximal cost-effectiveness.

In the mixed aetiology cohort, which approximates the decision framework if a single strategy is to be adopted across all aetiologies, surveillance of any kind can only be recommended if willingness to pay approaches £30 000 per QALY. At this level, 6-monthly AFP-triage appears to be the most cost-effective surveillance protocol, and remains the foremost option until willingness to pay reaches very high levels. This analysis suggests that society would have to be prepared to spend nearly £70 000 per QALY gained before the most effective strategy – 6-monthly AFP+US – could confidently be assumed to provide best value for money.

## DISCUSSION

### Summary of main findings

Our analysis suggests that, in patients with cirrhosis, surveillance strategies for HCC are effective, and can often be considered cost-effective. The most effective strategy for a mixed aetiology cohort of individuals with cirrhosis is AFP assay combined with US imaging on a 6-monthly basis. However, when costs are taken into account, using AFP as a triage step may be preferable. Surveillance is much more likely to be cost-effective in those with HBV-related cirrhosis, while surveillance of people with ALD-related cirrhosis appears least economically efficient.

### Interpretation of findings

According to our simulation, the economic efficiency of different surveillance strategies can be expected to vary substantially according to cirrhosis aetiology. As a result, the most efficient mode of resource allocation, from a purely decision-analytic viewpoint, would be to offer surveillance of differing intensity to each subgroup. However, apart from the practical complications of establishing different recall pathways for different patient groups, concerns might also be raised about the ethical implications of this approach. For instance, one-way sensitivity analysis suggested that the particularly good value offered in the HBV group may be substantially due to the younger age of the cohort. By implication, then, there may be further subgroups of individuals with HCV and ALD, diagnosed with cirrhosis at a younger age, in which more intensive surveillance might be particularly cost-effective.

Our results also suggest that reports of the AFP test's demise (Sherman, 2001) may be exaggerated, particularly if one adopts a cost-effectiveness perspective. We believe one reason for this is that previous authors may have failed to account fully for tumour size in their analyses. Although the AFP test has been found to be relatively insensitive for detecting HCC in general (Trevisani *et al*, 2001), it has the substantial advantage that its sensitivity is only weakly correlated with tumour size, meaning that it should be a valuable aid to the detection of some of the smallest, most easily treated tumours. According to the evidence used in our model, 65% of tumours less than 2 cm in diameter secrete 20 ng ml^−1^ or more of AFP (although this figure may be an overestimate, with the true proportion around 46%; Farinati *et al*, 2006). In contrast, our model is configured to simulate US sensitivity of only 10.7% for the smallest category of tumour (Bennett *et al*, 2002). Although this is at the pessimistic end of the range of available evidence, it should not be seen as an outlying estimate. Every study that has used an optimal reference standard (explant pathology) to investigate the diagnostic capabilities of US, in this setting, has reported disappointing sensitivity for the tumours ⩽2 cm in diameter: 13.8% (Kim *et al*, 2001), 22.2% (Rode *et al*, 2001) and 30.0% (Liu *et al*, 2003). Even if one was to adopt the most positive estimate available, all robust evidence suggests that US is less sensitive than AFP assay for the detection of the smallest tumours.

Additionally, AFP is a very cheap test and, inevitably, this is a crucial consideration from a cost-effectiveness perspective. The fact that current guidelines do not recommend using AFP screening may reflect the poor quality of current evidence or a lack of explicit attention to cost-effectiveness considerations during the development of clinical guidelines. However, a strategy led by one single diagnostic modality will always run the risk of serial false-negative findings, and this setting provides a good example (some tumours never secrete AFP and will therefore never be detected using an AFP-led approach and, equally, some tumours will infiltrate diffusely and resist US detection).

Because tumour growth rate had a clear influence on cost-effectiveness in the one-way sensitivity analysis, we investigated this factor further. We performed stratified scenario analyses, simulating three mixtures of slow-growing and fast-growing tumours, instead of applying one average growth rate throughout the model. While the combination of growth rates had an impact on the effectiveness and cost-effectiveness of surveillance (the more fast-growing HCCs, the more cost-effective surveillance becomes), the incremental relationship between surveillance strategies was preserved. The only practical implication of these additional findings is to suggest that, if the true mix of HCCs features a preponderance of slow-growing tumours, it may not be cost-effective to offer any surveillance strategy at 6-monthly intervals.

### Strengths of the evaluation

This is the first analysis of the effectiveness and cost-effectiveness of surveillance of cirrhosis for HCC in the UK NHS. Comprehensive literature searches were conducted to inform the model parameters, wherever possible choosing data either derived from the UK population or most likely to be applicable to the UK population.

Extensive exploration of model structures and uncertainty suggest that our model more appropriately captures the disease and surveillance process and impacts than previous studies in this field. While we have assumed that decompensated cirrhosis is irreversible, we showed in one-way sensitivity analysis that our results are not greatly influenced by alterations in the annual rate of progression from compensated to decompensated cirrhosis. From this, it is safe to infer that allowing some regression from decompensated back to compensated cirrhosis would not significantly alter our main results. By accounting for the substantial differences in age-related incidence, natural history and response to treatment that exist according to cirrhosis aetiology, we predict that different approaches to surveillance may be justified according to different causes of cirrhosis.

### Limitations of the evaluation

First, there is very little published evidence on which to base many of the parameter estimates for the model, and few data originate within the United Kingdom. This was particularly apparent for defining US performance. Second, as the primary focus of this evaluation was the effectiveness and cost-effectiveness of surveillance, we have used a simplified approach to modelling treatment in which OLT and resection are the only curative options available. We performed limited modelling of nonsurgical/ablative therapies as the evidence at the time of the analysis was inconclusive (Di Bisceglie, 2005). Recent evidence, predominantly from Asia (Izumi *et al*, 2007; Peng *et al*, 2007; Zytoon *et al*, 2007), suggests that such therapies may improve survival in patients with small tumours. If similarly promising findings could be shown in the United Kingdom, we would expect widespread adoption of such techniques to have beneficial cost-effectiveness implications, since a minimally invasive approach is much cheaper than OLT, and might also be expected to have less negative impact on quality of life. Third, we have assumed that entry to the surveillance programme is confined to those more likely to be considered eligible for the available curative treatment options (OLT or resection) and therefore assumes that high-risk activity (excessive alcohol consumption, intravenous drug use and so on) has ceased, and this may not be realistic. We have also assumed that the three cirrhosis aetiologies are mutually exclusive and acknowledge that many people develop cirrhosis as a result of multiple causes.

### Comparison with other studies

There are no other studies published studies from Europe. We identified three comparable studies from our literature searches (Arguedas *et al*, 2003; Lin *et al*, 2004; Patel *et al*, 2005); all were conducted in the United States and simulated HCV-related cirrhosis only. In these studies, 6-monthly surveillance using AFP and US produced utility gains of between 0.23 and 0.49 QALYs, compared with no surveillance, giving ICERs of between $24 500 and $46 600 per QALY gained. Apart from obvious dissimilarities in input values and assumptions – reflecting the different populations and health-care systems simulated – there are substantial differences in the structures of the models. Crucially, ours is the only model to have accounted for tumour size in simulating the sensitivity of surveillance.

### Implications for future research

Further research is required in the following areas (i) further modelling studies using alternative modelling methods such as individual patient sampling techniques could be used to account for heterogeneity in the patient population, so that factors such as tumour growth rate, tumour characteristics and the variability in individual patients’ serial test results could be assessed. Such methods could also be used to assess the optimal surveillance strategy, the optimal surveillance interval and the effects of surveillance on waiting lists for OLT; (ii) further modelling studies could also investigate innovative surveillance strategies not currently undertaken in clinical practice (e.g., alternating AFP and US investigations at 6- or 12-monthly intervals, or measuring change in AFP levels following serial tests rather than absolute levels with fixed cutoff points); (iii) further empirical and modelling analysis of the impact of age at diagnosis of cirrhosis on the effectiveness and cost-effectiveness of surveillance; (iv) empirical evaluation of newer imaging techniques (e.g., contrast-enhanced US) to detect HCCs; and (v) assessment of the effectiveness and cost-effectiveness of surveillance in other aetiologies (e.g., nonalcoholic fatty liver disease) and using other curative treatment options (e.g., ablative techniques).

## Figures and Tables

**Table 1:**
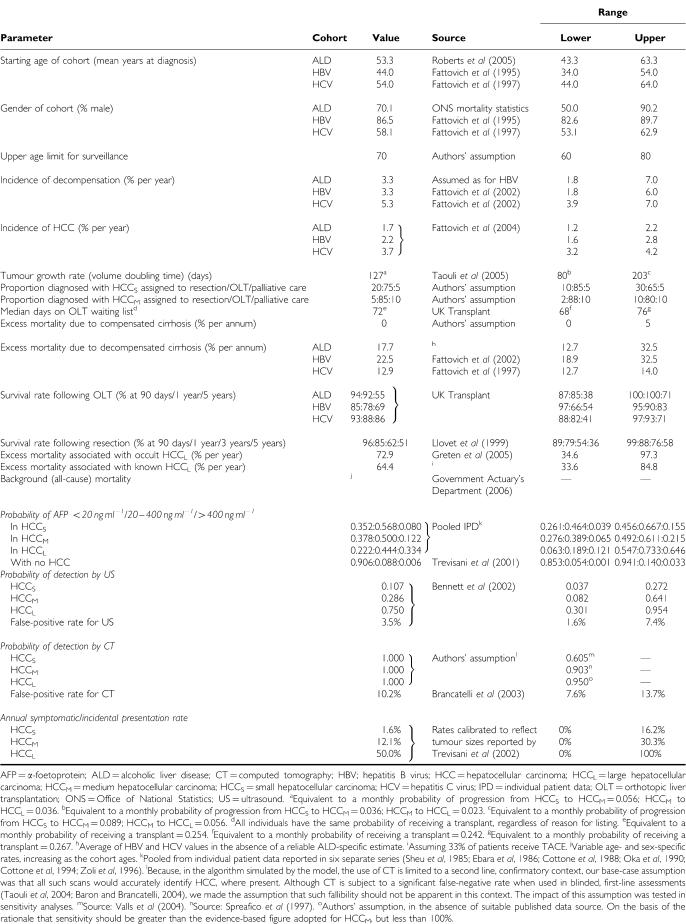
Model parameters: transition probabilities defining natural history, surveillance and therapy

**Table 2:**
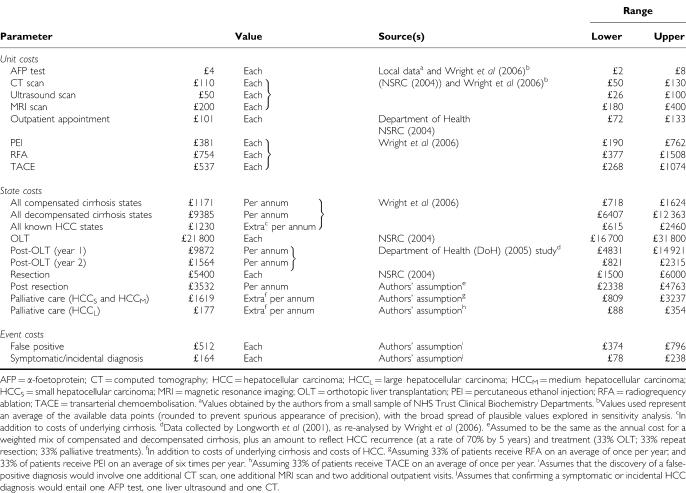
Model parameters: costs

**Table 3:** Model parameters: utility values

				**Range**	
**Health state**	**Markov states in which applied**	**Value**	**Source**	**Lower**	**Upper**
Compensated cirrhosis	All compensated cirrhosis states (±known or occult HCC_S_ or HCC_M_, including patients on the OLT waiting list)	0.75	Chong *et al* (2003)	0.66	0.83
Decompensated cirrhosis	All decompensated cirrhosis states (±known or occult HCC_S_ or HCC_M_, including patients on the OLT waiting list)	0.66	Chong *et al* (2003)	0.46	0.86
HCC	Terminal HCC_L_	0.64	Chong *et al* (2003)	0.44	0.86
Month of OLT	OLT (month of)	0.50	Authors’ assumption	0.30	0.60
Post-OLT (year 1)	Post-OLT (year 1)	0.69	Ratcliffe *et al* (2002)	0.64	0.74
Post-OLT (year 2+)	Post-OLT (year 2 onwards)	0.73	Ratcliffe *et al* (2002)	0.67	0.78
Resection	Resection (month of)	0.50	Authors’ assumption	0.30	0.60
Post resection	Post resection	0.73	a	0.62	0.84

HCC=hepatocellular carcinoma; HCC_L_=large hepatocellular carcinoma; HCC_M_=medium hepatocellular carcinoma; HCC_S_=small hepatocellular carcinoma; OLT=orthotopic liver transplantation.

a Weighted average of the values adopted for compensated and decompensated cirrhosis.

**Table 4:** Lifetime effectiveness of surveillance

	**No surveillance**	**Annual AFP-triage**	**Annual US**	**Annual AFP+US**	**6-monthly AFP-triage**	**6-monthly US**	**6-monthly AFP+US**
% with operable HCC	5.1%	11.9%	11.7%	13.5%	15.3%	15.0%	16.9%
% HCC_S_ at diagnosis	0.3%	1.9%	1.5%	2.3%	3.1%	2.6%	3.7%
% HCC_M_ at diagnosis	2.1%	3.8%	4.1%	4.2%	4.2%	4.6%	4.4%
% getting OLTs	17.1%	19.1%	19.1%	19.2%	20.1%	20.0%	20.3%
% OLTs for known HCC	8.3%	20.3%	20.0%	23.2%	25.3%	24.9%	27.9%
% dying of HCC	19.9%	14.7%	14.9%	13.5%	12.0%	12.3%	10.8%
NNS to prevent 1 death^a^	—	19	20	15	13	13	11
% dead by age 75 years	69.3%	68.4%	68.5%	68.2%	68.0%	68.0%	67.8%
NNS to prevent 1 death^b^	—	114	117	93	78	79	68

HCC=hepatocellular carcinoma; HCC_L_=large hepatocellular carcinoma; HCC_M_=medium hepatocellular carcinoma; HCC_S_=small hepatocellular carcinoma; NNS=number needed to be under surveillance; OLT=orthotopic liver transplantation.

a NNS to prevent one death from HCC.

b NNS to prevent one ‘premature’ death (age <75 years).

**Table 5:** Cost–utility analyses

			**Incremental analysis**		
**Strategy**	**Cost (£)**	**Utility (QALYs)**	**Cost (£)**	**Utility (QALYs)**	**£/QALY (ICER)**
*ALD*					
No surveillance	£26 100	9.359			
Annual AFP-triage	£27 400	9.410	£1300	0.051	£24 800
Annual US	£27 700	9.410	Extendedly dominated		
Annual AFP+US	£28 100	9.422	Extendedly dominated		
6-monthly AFP-triage	£28 200	9.433	£800	0.024	£35 500
6-monthly US	£28 800	9.434	Extendedly dominated		
6-monthly AFP+US	£29 200	9.445	£1000	0.011	£88 000
					
*HBV*					
No surveillance	£29 600	10.858			
Annual AFP-triage	£31 700	11.069	£2100	0.211	£10 200
Annual US	£32 100	11.066	Dominated		
Annual AFP+US	£32 700	11.119	Extendedly dominated		
6-monthly AFP-triage	£33 000	11.168	£1300	0.099	£12 700
6-monthly US	£33 600	11.164	Dominated		
6-monthly AFP+US	£34 200	11.216	£1300	0.048	£26 800
					
*HCV*					
No surveillance	£27 600	8.087			
Annual AFP-triage	£29 500	8.172	£1900	0.085	£22 200
Annual US	£29 700	8.172	Extendedly dominated		
Annual AFP+US	£30 300	8.193	Extendedly dominated		
6-monthly AFP-triage	£30 600	8.212	£1100	0.040	£27 600
6-monthly US	£31 000	8.213	Extendedly dominated		
6-monthly AFP+US	£31 600	8.232	£1000	0.020	£50 400
					
*Mixed aetiology*					
No surveillance	£26 900	9.021			
Annual AFP-triage	£28 400	9.096	£1500	0.075	£20 700
Annual US	£28 800	9.096	Dominated		
Annual AFP+US	£29 200	9.114	Extendedly dominated		
6-monthly AFP-triage	£29 400	9.131	£1000	0.035	£27 600
6-monthly US	£29 900	9.131	Dominated		
6-monthly AFP+US	£30 400	9.148	£1000	0.017	£60 100

AFP=*α*-foetoprotein; ALD=alcoholic liver disease; HBV; hepatitis B virus; HCV=hepatitis B virus; ICER=incremental cost-effectiveness ratio; QALY=quality-adjusted life-year; US=ultrasound.

Discount rate of 3.5% per annum applied to all costs and benefits.
